# Simulation-Based Evaluation of the Performances of an Algorithm for Detecting Abnormal Disease-Related Features in Cattle Mortality Records

**DOI:** 10.1371/journal.pone.0141273

**Published:** 2015-11-04

**Authors:** Jean-Baptiste Perrin, Benoît Durand, Emilie Gay, Christian Ducrot, Pascal Hendrikx, Didier Calavas, Viviane Hénaux

**Affiliations:** 1 Unité Epidémiologie, Agence nationale de sécurité sanitaire de l’alimentation, de l’environnement et du travail—Laboratoire de Lyon, Lyon, France; 2 Unité Epidémiologie, Agence nationale de sécurité sanitaire de l’alimentation, de l’environnement et du travail—Laboratoire de Santé Animale, Maisons-Alfort, France; 3 Unité Epidémiologie animale, UR346, INRA, St Genès Champanelle, France; 4 Unité Coordination et appui à la surveillance, Agence nationale de sécurité sanitaire de l’alimentation, de l’environnement et du travail—Laboratoire de Lyon, Lyon, France; Univ. Prince Edward Island Atlantic Veterinary College, CANADA

## Abstract

We performed a simulation study to evaluate the performances of an anomaly detection algorithm considered in the frame of an automated surveillance system of cattle mortality. The method consisted in a combination of temporal regression and spatial cluster detection which allows identifying, for a given week, clusters of spatial units showing an excess of deaths in comparison with their own historical fluctuations. First, we simulated 1,000 outbreaks of a disease causing extra deaths in the French cattle population (about 200,000 herds and 20 million cattle) according to a model mimicking the spreading patterns of an infectious disease and injected these disease-related extra deaths in an authentic mortality dataset, spanning from January 2005 to January 2010. Second, we applied our algorithm on each of the 1,000 semi-synthetic datasets to identify clusters of spatial units showing an excess of deaths considering their own historical fluctuations. Third, we verified if the clusters identified by the algorithm did contain simulated extra deaths in order to evaluate the ability of the algorithm to identify unusual mortality clusters caused by an outbreak. Among the 1,000 simulations, the median duration of simulated outbreaks was 8 weeks, with a median number of 5,627 simulated deaths and 441 infected herds. Within the 12-week trial period, 73% of the simulated outbreaks were detected, with a median timeliness of 1 week, and a mean of 1.4 weeks. The proportion of outbreak weeks flagged by an alarm was 61% (i.e. sensitivity) whereas one in three alarms was a true alarm (i.e. positive predictive value). The performances of the detection algorithm were evaluated for alternative combination of epidemiologic parameters. The results of our study confirmed that in certain conditions automated algorithms could help identifying abnormal cattle mortality increases possibly related to unidentified health events.

## Introduction

Faced with the challenge of detecting emerging health threats by traditional epidemiological surveillance systems, there has been an increasing interest in syndromic surveillance. Such systems rely on the real-time collection, analysis, interpretation, and dissemination of health-related data to enable the early identification of the impact (or absence of impact) of potential human or veterinary public health threats that require effective public and/or animal health action [1]. Data used in syndromic surveillance are non-specific health indicators including clinical signs or symptoms that are collected for purposes other than surveillance. Most public health agencies of developed countries have collected for a long time human mortality data in order to monitor chronic diseases, plan public health programs, or evaluate their efficiency [2]. These purposes did not require data to be collected and analyzed in a timely manner. But after the heat wave that hit Europe in 2003, several countries implemented systems aimed at rapidly detecting sudden increases of human mortality. A European project, called EuroMoMo (European Mortality Monitoring) and gathering 20 Member States, was notably launched in 2008 to promote and implement weekly surveillance of mortality in human population at the European level, in order to possibly detect health threats such as major epidemics, extreme weather events, and deliberate or accidental release of biological or chemical agents [2,3]. In animal health, mortality surveillance systems dedicated to early warning are uncommon although many data are routinely collected, notably through national registers and rendering plants [4]. These huge databases, which are theoretically comprehensive and rapidly updated, have regularly been used for retrospective analyses [5–7], but further developments are required for implementing prospective syndromic surveillance systems.

Cattle mortality data that are routinely collected in France were used to monitor cattle population health and report unusual mortality patterns. Retrospective studies were first carried out to model the cattle mortality baseline in France [8], assess the impacts of the 2008 bluetongue disease epidemics [9], and quantify the impact on the cattle population of the 2003 and 2006 heat waves [5]. These studies confirmed the quality of the two databases available, the National cattle register (NCR) and the Fallen stock data interchange (FSDI), and their potential suitability for surveillance [6]. However, the most adequate methods to prospectively analyse the continuous flow of cattle mortality data and rapidly detect anomalies still needed to be identified and evaluated.

Many methods for detecting outbreaks have already been considered for epidemiological surveillance, most being based on the detection of anomalies in time series [10,11]. Common methods for outbreak detection include statistical tests for space-time interaction, cumulative sum methods, and scan statistics [12]. The choice of a particular algorithm to detect unusual signals (e.g., abnormal increases of deaths) in the evolution of an health indicator remains difficult since their performances greatly vary according to factors related to the signal to be detected (time of onset, magnitude, space and time progression), the baseline of the indicator monitored (incidence, shape, and variation) and the system itself (data source, data quality, processing frequency). Scan statistic methods may be used in temporal, spatial, or spatio-temporal settings, depending on whether the times and/or locations of cases are known. The simplicity of this approach, its ability to deal with large volume of data, and the ease of understanding and interpretation of results [12] make that this methodology is frequently used in both human and animal health applications.

Evaluating the performance of outbreak detection algorithms is thus essential to assess their efficiency in detecting early and accurately signals that indicate changing health patterns [13]. Two main types of evaluation can be considered: epidemiological and simulation approaches [14]. The epidemiological approach is based on authentic outbreaks whose occurrence is determined by objective criteria derived from epidemiological investigations or by expert judgment. This approach can be carried out prospectively when the surveillance system is operational, by describing which naturally occurring outbreaks are detected and missed [15]. In the absence of historical data containing known examples of events a surveillance system is designed to detect, evaluation of the detection system’s performances is only possible using simulation [16]. In contrast to studies based on authentic datasets which can only estimate performances for a particular event in certain conditions, simulation studies are flexible and allow a more comprehensive characterization of detection properties since the number and timing of cases added to the baseline is perfectly known [17].

The objective of our study was to evaluate the performances of a method that we designed for detecting abnormal increases in the cattle mortality baseline in France. The method that we evaluated was a combination of temporal regression and spatial cluster detection which allows identifying, for a given week, clusters of spatial units showing an excess of deaths in comparison to their own historical fluctuations. The ability of this approach to detect abnormal increases of mortality caused by infectious outbreaks was evaluated through a simulation study. First, we simulated 1,000 outbreaks causing extra deaths according to a model mimicking the spreading patterns of an infectious disease and injected these disease-related extra deaths in a dataset of bovine mortality in France. Second, we applied our algorithm on each of the 1,000 semi-synthetic dataset obtained by simulation to identify clusters of spatial units showing an excess of deaths considering their own historical fluctuations. Third, we verified whether the clusters identified by the algorithm did contain simulated extra deaths (and inversely) in order to evaluate the ability of the algorithm to identify unusual mortality clusters caused by an outbreak. Fourth, we evaluated the performances of the algorithm in detecting simulated outbreak mortality based on alternative disease scenarios, through a sensitivity analysis. Finally, we discussed the results and the relevance of the evaluation approach, and the next steps to achieve the implementation of an automated cattle mortality surveillance system.

## Material and Methods

Data were administrated with Toad for MySQL [18], analyzed with R [19], and scan statistics were computed with SatSCAN software [20]. The study was based on the cattle identification data and movement notifications—including deaths—recorded in the French National cattle registration (NCR) from 2005 to 2010. We kept data from mainland France only and excluded French overseas territories.

### Creating the Baseline Dataset

First, we used data extracted from the NCR to compute the weekly number of cattle deaths and number of animals-days alive by herd using the algorithm described in Perrin et al. (2010) [9]. We aggregated daily mortality data to obtain weekly figures, as the exact dates of the movements registered in the NCR (including death dates) were not very reliable and not randomly distributed over the week. For example, the notified date of death is heavily influenced by the farmers’ behavior (e.g. level of herd surveillance intensity) and by the particular work organization of each fallen stock company (many farmers notify not the date they discover a cadaver but the date of its disposal by fallen stock companies, which usually do not collect cadavers during the week-end or bank holidays) [6]. The weekly number of animal-days alive was computed by summing the number of days each animal was present. Given this definition, three animal-days can represent one animal present on three days or three animals on one day, for example. Then, we divided mainland France in 1,125 regular hexagons of 25 km diameter using the R packages hexbin [21] in order to conduct the study at an intermediate spatial scale between the *department*, considered as too large, and the herd where the number of deaths is too low to run such analyses (a *department* is a French administrative and territorial division covering a mean surface area of 5,800 km^2^). Each cattle farm recorded in the NCR during the study period (272,712) was attributed to the hexagon that contained the centroid of its zip code; 3,906 farms (1.4%) were not attributed to a hexagon due to missing zip code. Finally the number of deaths and number of animal-days alive were aggregated by week and hexagon. As a result, for each of the 1,125 hexagons, we obtained a time series of weekly mortality rate from 2005-01-01 to 2010-02-01. The period before 2008-11-03 (196 weeks, that is 3.8 years) was kept as the “calibration period” for the temporal anomaly detection methods whereas the 65-week period from 2008-11-03 to 2010-02-01 (1.3 years) was used as the “test period”, during which we injected simulated increases of mortality and applied our algorithm to detect them (see later).

The 65-week dataset contained atypical observations whose nature was unknown (i.e. real increases in mortality caused by health events, non-health events, or artifacts). Keeping these extreme death counts would have reduced the performances of the method by increasing the rate of false alarms, whereas we don't actually know if these extreme counts are signals we want to detect (true alarms) or not (false alarms). To correct these atypical observations we applied to each time series an automated method proposed by Fried (2004) [22] based on a robust regression functional for local approximation of a linear trend in a moving time window. Rousseeuw and Croux’s scale estimator was applied to the residuals to approximate the variability in the window [22]. Counts that exceeded the level plus three times the scale were considered as outliers and replaced by the current level estimate. Time series filtering was conducted using the R “robfilter” package [23].

### Simulating and Injecting Disease-Related Deaths in the Baseline Dataset

Extra deaths were simulated according to a scenario of an infectious disease that spreads rapidly in the cattle population and causes moderate mortality and rapid recovery in infected herds. Each simulation began with a random sampling of a farm among the French cattle farms and a date between 2008-11-03 and 2009-11-04 (the test period ending 12 weeks later). The spread of the outbreak was then simulated during a maximum of 12 weeks or until it reached 15,000 deaths, according to a model taking into account both within-herd and between-herd dissemination of the disease (simplified from [24,25]). The details of this model and the values of its parameters are given in S1 File.

Within-herd dissemination was modelled using a SIR model, which gave the number of Susceptible, Infected, Recovered and dead animals by day after the disease was introduced in a herd. We considered a disease with a reproduction ratio R_0_ of 5, a daily recovery rate of 0.14 and a daily mortality rate of 0.03 (i.e. a probability of dying of 2.9%; S1 File); these parameters are comparable to those reported for foot-and-mouth disease [26,27]. NCR data were used to determine the number of susceptible animals in herds during the 12 weeks of the simulation; all animals were considered to be equal, whatever their age and breed.

Between-herd dissemination was modelled by taking into account both spatial location of herds and the exchanges of live cattle between herds. Since exact geographic coordinates of French herds were unknown, they were drawn at random inside their zip code (once for all the simulations). The probability that an infected herd infects a susceptible neighbouring herd, through direct transmission in pastures, was defined by the transmission kernel, which determines the probability of pathogen transmission from infected to uninfected farms as a function of inter-farm distance and a cattle-specific biosecurity parameter limiting disease transmission (see S1 File). The probability that an infected herd transmits the disease through the sale of an animal was defined by the disease prevalence (i.e. the proportion of infected animals) in the origin herd at that time. Movement notifications recorded in the NCR were used to identify the date and destination herd of cattle sold by infected herds during the test period.

As a result, for each simulation, the model provided the identification number of the infected herds and their date of infection, and the number of extra deaths caused by the disease by date and infected herd. Weeks during which at least one simulated death was injected were defined as “Outbreak weeks”.

A total of 1,000 outbreaks were simulated. For each of them, the simulated deaths were aggregated by hexagon and week and injected in the baseline dataset described previously. We obtained in this manner 1,000 semi-synthetic datasets containing an abnormal increase of deaths caused by a simulated outbreak.

### Method Used to Identify Abnormal Increase of Mortality

The spatial scan method to identify clusters of hexagons presenting excess deaths in comparison to their own historical fluctuations was conducted in two steps: first, baseline of weekly expected count of cattle deaths in each hexagon were inferred from the time-series of previous counts (calibration dataset); second, spatial scan analysis was applied to compare predicted (historical) to observed (real and simulated) number of deaths and identify clusters of increased counts.

#### Poisson regression models

In each hexagon, we fitted a Poisson model (log link) on the calibration period, taking into account the number of calvings (which has been demonstrated to influence the mortality rate [6,8]) and a month effect as covariates, and a trend that was systematically removed when not significant (p-value < 5%). An overdispersion parameter was added when the overdispersion of the errors of the Poisson models was tested significant, using the R poisgof() function [28]. To reduce the effect of mortality aberrations in the calibration period, we performed a second round of estimation of the model, weighting the observed values by the inverse of their residuals, according to the procedure proposed in Farrington et al. (1996) [29]. Reweighting was conducted using the R “surveillance” package [30]. Not correcting for these extreme high values would have resulted in higher baseline mortality and consequently would have reduced the ability of the detection algorithm to detect simulated mortality. Finally, we used the models to predict the baseline number of deaths by week and hexagon during the test period under the hypothesis of the absence of any particular health event.

#### Spatial scan statistics

Spatial regions (clusters of hexagons) showing excess mortality in comparison to their own historical fluctuations were tracked each week of the test period using spatial scan statistics computed with the SaTScan software [20].

The scan statistic was based on a Poisson model that requires information on the number of cases and the size of the population in each location (hexagon). With this model, raw population numbers can be replaced by covariate adjusted values for the expected number of cases estimated by standard statistical regression [31]. For our algorithm, the number of cases, referred to as “observed number of deaths”, corresponded to the number of real deaths plus the number of simulated deaths (if any). Population numbers were replaced by the “expected number of deaths”, i.e. the number of deaths predicted by the Poisson models adjusted for each hexagon. The advantage of combining the Poisson regression models and the spatial scan statistics (rather than using only spatial scan statistics) is to focus the detection only on clusters that are unusual, and to ignore those that are actually seasonal and repeated each year. Indeed, it has been showed that mortality rates in France are seasonal and that their seasonality varies from one department to another [6]. As a consequence, the mortality baseline includes seasonal cluster of mortality. Using spatial or space-time scan statistics on the data would have led to detect known and seasonal clusters of mortality.

SaTScan detects clusters of cases by gradually scanning a circular region (window) across space, recording the number of observed and expected observations inside the window in each hexagon. Circles of varying radii were sequentially centred on the centroid of each hexagon and the ratio of cases to controls was computed for the areas inside and outside each circle. The maximum circle size was set to 20% of the cattle population under surveillance. The method tests the null hypothesis H_0_ (i.e. no cluster) against the set of alternative hypotheses H_1_(S) (each representing a cluster in region S). SaTScan test statistic is the likelihood ratio, which corresponds to the likelihood of the data under the alternative hypothesis H_1_(S) divided by the likelihood of the data under the null hypothesis H_0_ [32]. The window with the maximum likelihood ratio was the most likely cluster.

SaTScan calculated the p-value using a combination of Monte-Carlo hypothesis testing and the Gumbel approximation [33] based on 999 random replications of the data set generated under the null hypothesis: if the maximum likelihood ratio calculated for the most likely cluster in the real data set is high compared to the maximum likelihood ratios calculated for the most likely clusters in the random data sets, that is evidence against the null hypothesis and for the existence of the cluster. For each week of the test period, the scan statistics analysis gave: the list of the hexagons included in the cluster identified, the p-value of the cluster (i.e. probability that the cluster results from a random distribution of cases), and the relative risk (RR) of mortality inside the cluster in comparison to outside.

### Performances Evaluation

To evaluate the performances of the algorithm in detecting the abnormal increase of disease-related mortality, we applied it on each of the 1,000 semi-synthetic datasets and verified if the hexagons clustered by our algorithm were those that contained the simulated extra deaths.

#### Alarm definition

For each week of the test period, the cluster analysis led either to a true alarm, a false alarm or no alarm. A true alarm was defined as the identification of a cluster containing at least one hexagon with one or more simulated deaths, referred to as “infected hexagons”. A false alarm was defined as the identification of a cluster containing no hexagon with simulated deaths. There was no alarm when the algorithm did not identify a cluster of hexagons.

#### Calibrating the algorithm to reach a given specificity

We considered as clusters only windows whose p-value was under a predefined maximal threshold (α) and relative risk above a predefined minimal threshold (RR_c_). Values of α and RR_c_ were set empirically so as to constrain the alarm rate in the entire country to remain under 4 false alarms per semester on the baseline dataset (without injecting any extra deaths), which we considered as a sustainable threshold for veterinary health services to investigate each alarm.

#### Indicators estimated from each trial

For each of the 1,000 trials, we recorded the p-value, relative risk and hexagons included in the clusters identified on each week of the test period. We calculated eight performance indicators at the end of each trial, defined in Table 1. Specificity was not evaluated since it was constrained by the study design. For sensitivity, positive and negative predictive values, two types of indicators were computed using two different units: week and hexagon-week. The first, type (a), was based on the number of true/false alarms produced over the test period. The second, type (b), was based on the number of hexagons-weeks included/not included in clusters during the test period. This second type of estimates (b) was proposed to complement the evaluation and estimate which proportion of infected hexagons was actually detected.

**Table 1:** Performance indicators stored from each trial.

Indicator		Definition
Success	-	Success equals 1 if there is at least one true alarm during the trial, 0 otherwise
Timeliness	-	Number of weeks elapsed between the first simulated death and the first true alarm
Sensitivity_a_ *	Se_a_	Proportion of weeks with an alarm among the outbreak weeks
Positive predictive value_a_ *	PPV_a_	Proportion of true alarms among the alarms
Negative predictive value_a_ *	NPV_a_	Proportion of weeks with no simulated deaths among the weeks without alarm
Sensitivity_b_ *	Se_b_	Proportion of hexagon-weeks included in a cluster among hexagon-weeks infected
Positive predictive value_b_ *	PPV_b_	Proportion of hexagon-weeks infected among all the hexagon-weeks included in a cluster
Negative predictive value_b_ *	NPV_b_	Proportion of hexagon-weeks not infected among the hexagon-weeks not included in a cluster

*For sensitivity, positive and negative predictive values, two types of indicators were computed using two different units: week and hexagon-week.

The first, type (a), was based on the number of true/false alarms produced over the test period. The second, type (b), was based on the number of hexagons-weeks included/not included in clusters during the test period.

#### Indicators summarizing performances over the 1,000 trials

We estimated the global proportion of successes among all trials (i.e. the proportion of trials with at least one true alarm) and the cumulative proportion of successes, calculated as the proportion of success per week after the introduction of the disease. The distribution (mean, median, 10^th^ and 90^th^ quantiles) of performance indicators (Table 1) was computed over all trials, and for three groups of outbreaks classified according to the number of simulated deaths ([0–100],] 100–10,000],] 10,000-∞[).

### Analysis of Sensitivity Linked with Disease Parameters

A sensitivity analysis was performed to evaluate the influence of disease parameters on the performances of the algorithm. A total of 25 within-herd dissemination scenarios were simulated with five alternative combinations of reproduction ratio R_0_ (1, 3, 5, 7 and 9) and five daily mortality rates (0.01, 0.02, 0.03, 0.04 and 0.05). For each scenario, a total of 100 outbreaks were simulated. The performances of the algorithm in detecting simulated mortality was evaluated using the set of indicators previously described (Table 1). We then estimated the distribution of each performance indicator (mean, median, 10^th^ and 90^th^ quantiles) over the 100 trials for each scenario. Linear correlation coefficients (LCC) were used to measure the correlation between each model parameter (R_0_ and daily mortality rates) and median value of each performance indicator; the t-statistic was used to test the significance of the correlation coefficients.

## Results

### Creating the Baseline Dataset

As a result of the filtering, 5.1% of the weekly hexagonal counts in the initial dataset (3,749 from 73,120) were considered as outliers and trimmed, which resulted in the removal of 21,747 deaths from the 1,711,130 deaths observed in the real dataset over the test period (1.3%).

### Simulating and Injecting Disease-Related Deaths in the Baseline Dataset

Eight simulations started in a herd that could not be attributed to a hexagon due to a change in zip code and were thus removed. For the remaining simulations, the median duration of outbreaks was 8 weeks, the median number of simulated deaths was 5,627 and the median number of infected herds was 441. The median weekly average number of simulated deaths per infected herd was 1.6. Summarized results of outbreak simulations are presented in Table 2.

**Table 2:** Characteristics (median, IQ range) of 1000 simulated outbreaks (based on a SIR model with R_0_ of 5 and daily mortality rate of 0.03).

Descriptors	Q25	median	Q75
Duration (weeks) ^†^	6	8	10
Number of simulated deaths^†^	101	5,627	15,629
Proportion of simulated deaths* (%)	0.1	1.9	7.3
Number of infected herds	5	441	1,415
Proportion of infected herds* (%)	<0.001	0.21	0.66
Number of infected hexagons	1	26	57
Proportion of infected hexagons* (%)	0.1	2.3	5.1
Weekly average no. of simulated deaths per infected herd	1.2	1.6	2.8

^†^ The spread of the outbreak was simulated during a maximum of 12 weeks or until it reached ≥15,000 deaths.

* Proportion of simulated deaths, infected herds and hexagons were respectively calculated using the total number of deaths (real+simulated) that occurred during the course of the outbreak, and the total number of herds and hexagons under surveillance over this period.

**Fig 1:**
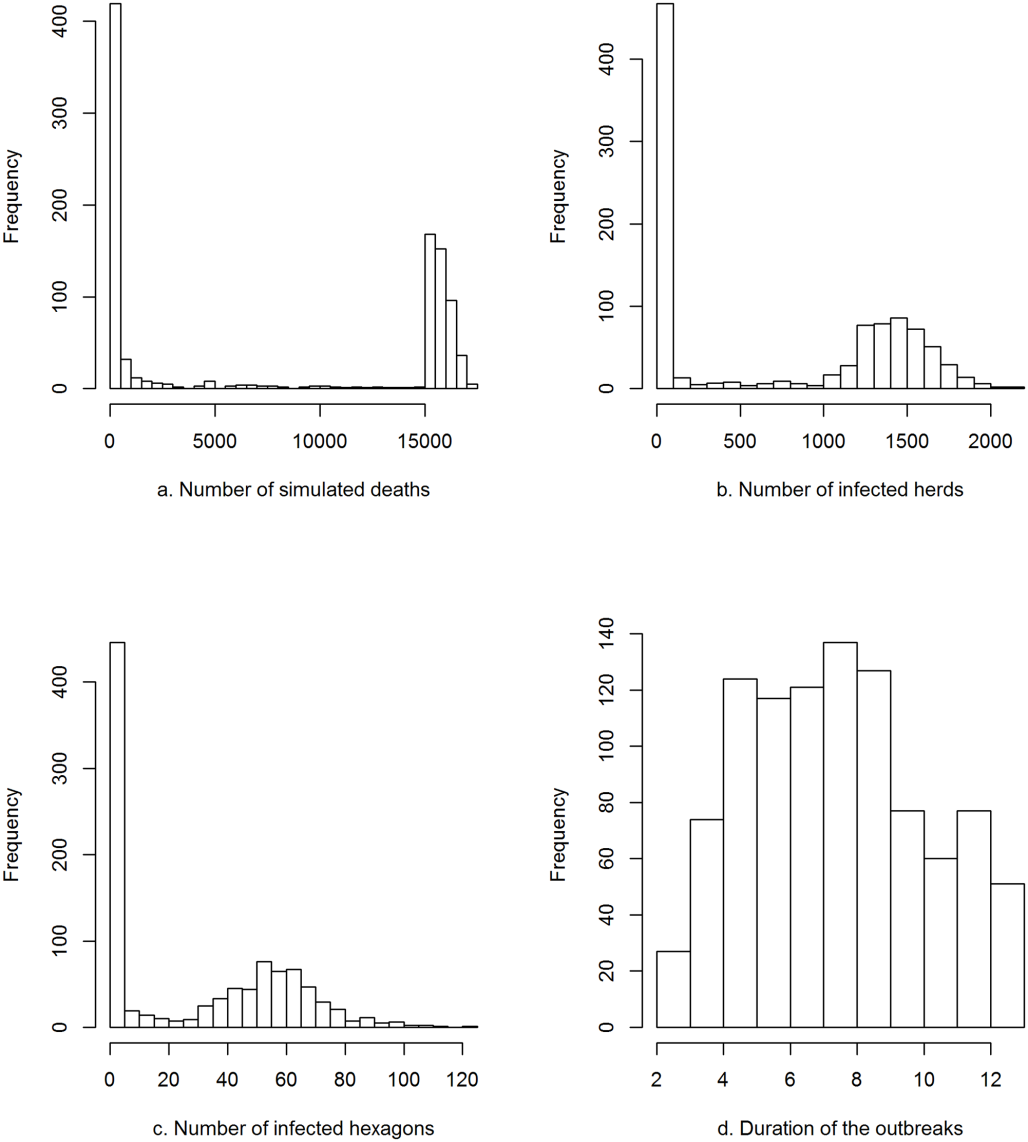
Characteristics of the 1000 outbreaks simulated with a reproduction ratio R_0_ of 5 and a daily mortality rate of 0.03. Distribution of the total number of simulated deaths (a), the number of infected herds (b) and infected hexagons (c), and the duration of the outbreak (d) among the 1,000 simulations. The spread of the outbreak was simulated during a maximum of 12 weeks or until it reached ≥15,000 simulated deaths.

The total number of simulated deaths and the number of infected herds and hexagons issued from the 1,000 simulations showed a bimodal distribution: 25% of the simulations ended in less than 100 simulated deaths and 48% in 15,000 or more (Fig 1). Among simulations, the number of infected herds peaked at [1–100] and [1,400–1,500], and the number of infected hexagons peaked at [1–5] and [50–55]. The duration of the outbreak was less variable among simulations with 63% of simulations lasting between 4 and 8 weeks (inclusive).

**Fig 2:**
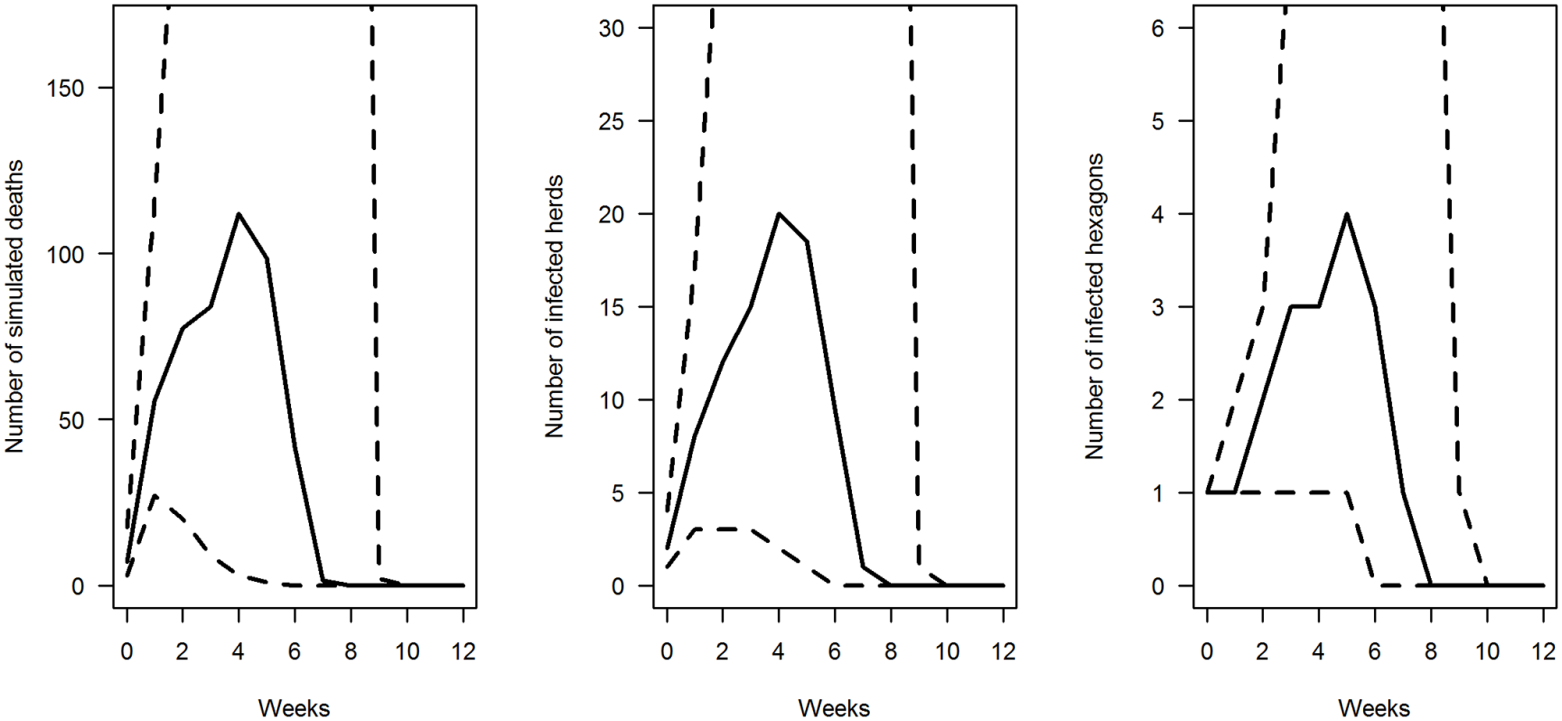
Characteristics over time of the 1000 outbreaks simulated with a reproduction ratio R_0_ of 5 and a daily mortality rate of 0.03. Median (solid line) and interquartile range (dashed lines) of the number of simulated deaths, infected herds, and infected hexagons by week over the 12 weeks following the start of the simulation, among the 1,000 simulations.

The median number of simulated deaths per outbreak showed a peak on the 4^th^ week after introduction of the disease (Fig 2). Features of the outbreaks varied dramatically between simulations: on week 4, the 25^th^ and 75^th^ quantiles of the number of simulated deaths were 3 and 912, respectively, while on week 6, the 25^th^ quantile was 0 but the 75^th^ quantile peaked at 1,937 deaths. Median number of infected herds and hexagons peaked on the 4^th^ and 5^th^ week after introduction of the disease, respectively.

### Evaluating the Performances

#### Calibrating the algorithm to reach a given specificity

To ensure a sufficient specificity (up to 4 false alarms per semester), only clusters with p-value under the threshold α = 0.001 and relative risk RRc > 1.25 were considered. With these constraints, the algorithm identified 9 clusters in the 65-week smoothed baseline dataset free from injected signals. Three successive weeks in March 2009 were included in a cluster consisting of 26, 23 and 8 hexagons, respectively, in east central France. Two successive weeks in April 2009 raised a false alert for 40 and 26 hexagons, respectively, in south France; another cluster in the same area occurred in May and included three hexagons. One false alert occurred in south-east France at the end of April 2009 and included 22 hexagons. One cluster impacted 63 hexagons in central France at the end of June. The last cluster occurred in central France at the end of December 2009 and included 24 hexagons. These clusters were not caused by extreme mortality values since we removed atypical observations, but rather by an increase in the baseline mortality. We collected no information on the real health events that occurred during the test period and could actually modify the mortality baseline, and thus we cannot determine if these false clusters are due to real health events or not. Thus the proportion of weeks without alarm was 86.2%, which corresponded to a biannual alarm rate of 3.6. Globally 0.3% of hexagon-weeks under surveillance (235/73,120) were included in a cluster.

#### Performance indicators

**Fig 3:**
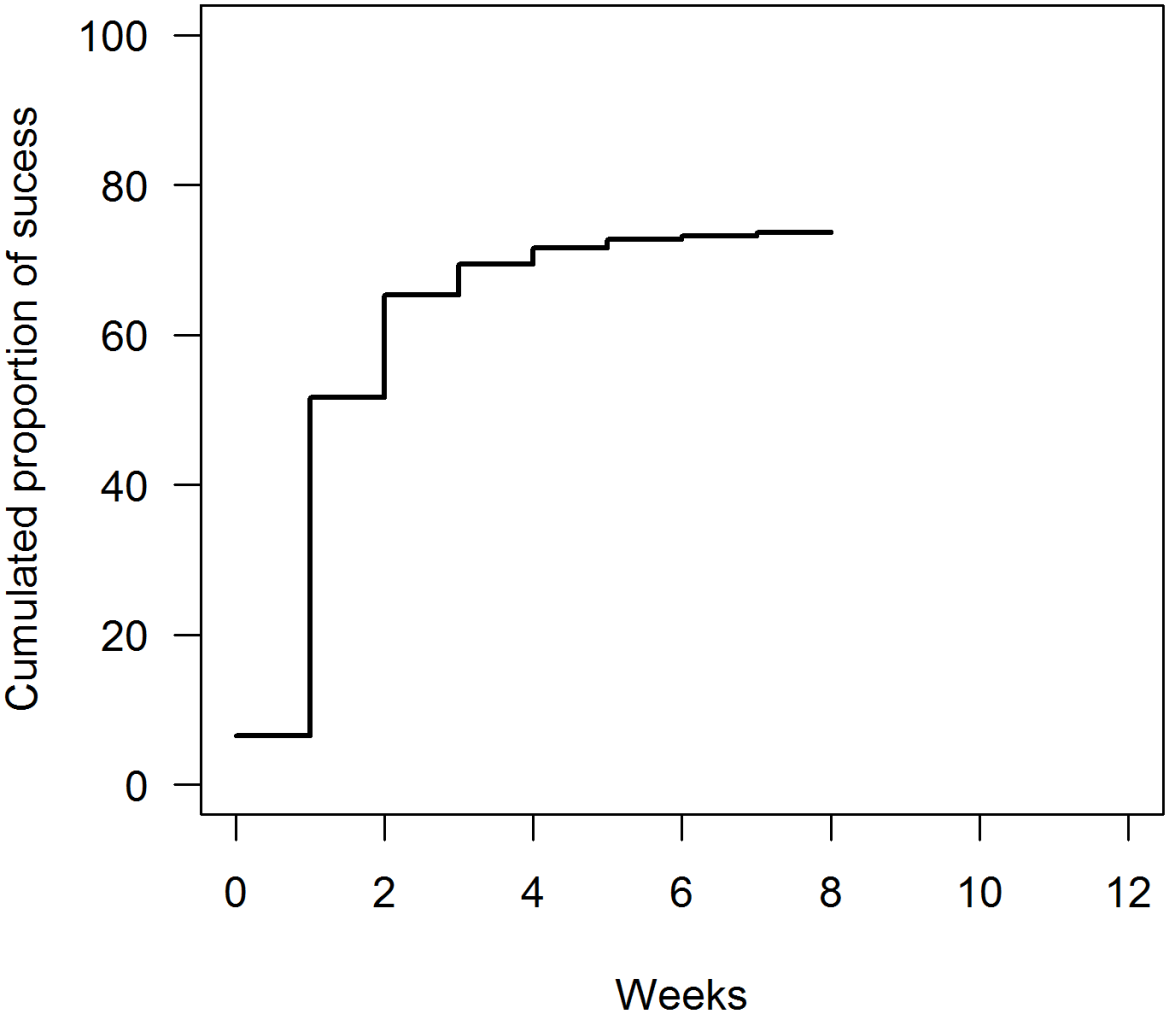
Cumulated proportion of success over the 12 weeks of the outbreak (n = 1,000 simulations).

The global success rate (i.e. the proportion of trials with at least one true alarm) was 73.2%. There was a very steep increase of success on the first week following the introduction of the disease. Success proportion increased from 6.5% (week 0, during which the disease was introduced) to 51.2% (week 1). On following weeks, the increase was slighter (Fig 3).

The median timeliness for detection among successful simulations was 1 week, with a mean of 1.4 weeks (Table 3). Median Se_a_ (proportion of outbreak weeks flagged by a true alarm) among the simulations was 61.5%. Median PPV_a_ (proportion of true alarms among alarms) was 35.7%, so about one in three alarms was a true alarm. The predictive positive value (PPV_a_) is constrained by the specificity of the system (constrained in our study to 4 false alarms per semester) and the duration of epizootics (median of 8 weeks; Table 2). Median NPV_a_ was equal to 94.6%, i.e. 94.6% of the weeks without alarm were not outbreak weeks. Mean Se_b_ (proportion of infected hexagons-weeks included in a cluster) was 13.0%. Median NPV_b_ (proportion of hexagon-weeks not infected among those not included in a cluster) was 99.9% and median PPV_b_ (proportion of infected hexagon-weeks among those included in a cluster) was 12.0%.

**Table 3:** Distribution among the 1,000 simulations of the performance indicators.

Indicators	Q10	median	Q90	mean
Timeliness (weeks)	1	1	3	1.4
Se_a_	0.0	61.5	90.0	49.6
PPV_a_	0.0	35.7	57.1	30.4
NPV_a_	89.3	94.6	100.0	94.6
Se_b_	0.0	12.5	26.0	13.0
PPV_b_	0.0	12.0	28.8	12.7
NPV_b_	99.8	99.9	100.0	99.9

**Fig 4:**
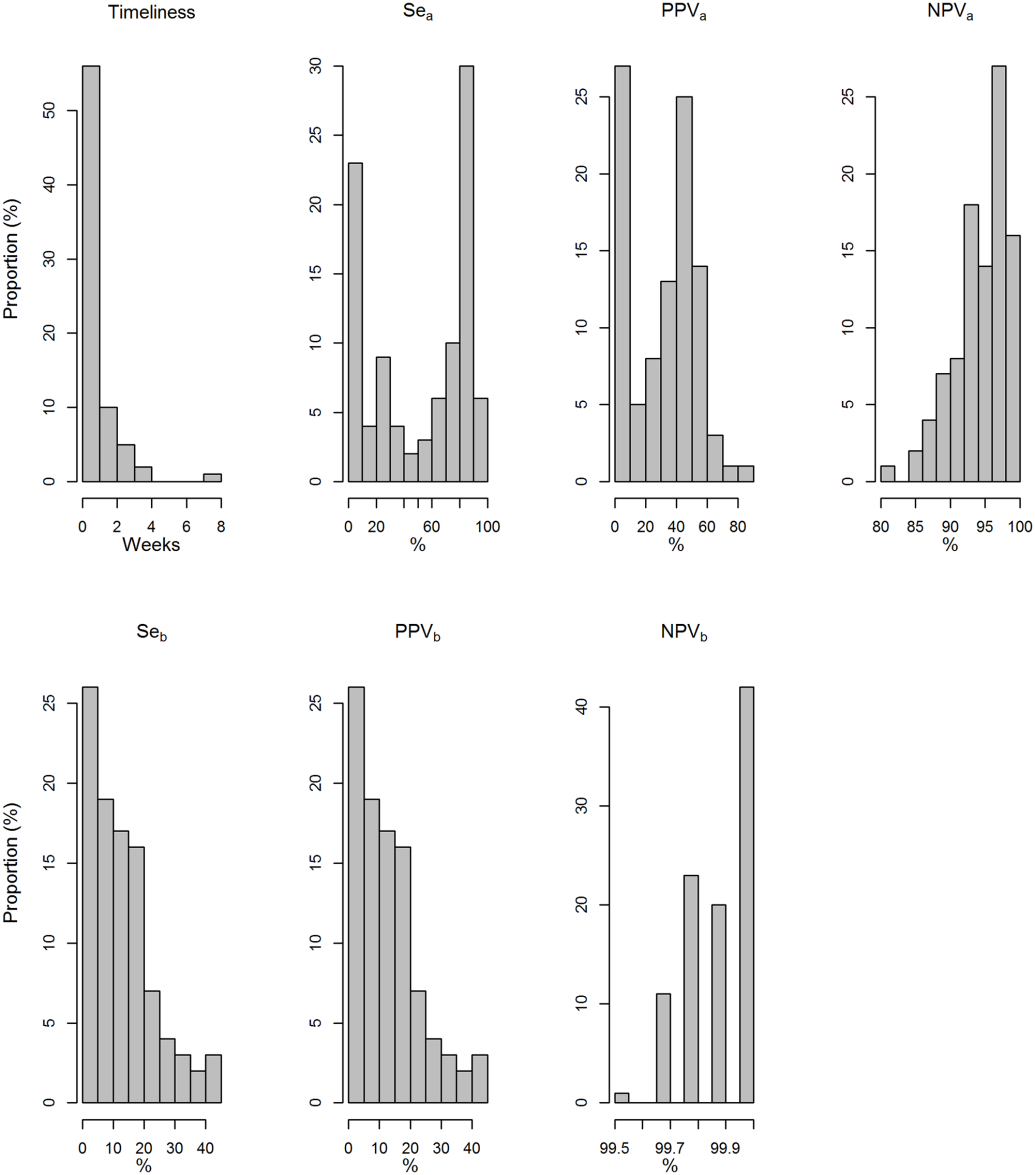
Distribution of the performance indicators among the 1,000 simulations.

The performance of the indicators varied among the simulations (Fig 4). For most indicators distribution was rather multimodal than unimodal.

#### Outbreak size at first alarm

Among the 73.2% simulations ending with a success (i.e. at least one true alarm during the trial), the median number of simulated deaths at the first alarm was 132 and the median number of infected herds was 16 (Table 4). The median average number of simulated deaths per infected herd at the week of the first alarm was 3.8 (Q10 = 2.0, Q90 = 7.7).

**Table 4:** Characteristics of the outbreak at first alarm (n = 1000 simulations based on a SIR model with R_0_ of 5 and daily mortality rate of 0.03).

Descriptors	Q10	Median	Q90	Mean
Number of simulated deaths	50	132	417	196
Proportion of simulated deaths* (%)	0.1	0.2	0.6	0.3
Number of infected herds	5	16	54	24
Proportion of infected herds* (%)	0.001	0.003	0.010	0.005
Number of infected hexagons	1	2	4	2.3
Proportion of infected hexagons* (%)	0.1	0.2	0.4	0.2

* Proportion of simulated deaths was calculated using the total number of deaths that occurred during the course of the outbreak as denominator. Proportion of infected herds and hexagons were based on the total number of herds and hexagons under surveillance during the course of the outbreak.

#### Effect of the outbreak size

The size of the outbreak (i.e. the number of simulated deaths) affected greatly the performances of detection. Among the 26% outbreaks that were not detected by the algorithm, the median overall number of simulated deaths at the end of the simulation was 49.5 and the median number of infected herds was 3. The algorithm detected 14.6% of the outbreaks causing less than 100 deaths whereas it detected 82.0 and 100% of the medium and large outbreaks, respectively (Table 5).

**Table 5:** Number of simulations, success rate, and median of the performance indicators according to the size of the simulated outbreaks.

Indicators	Small	Medium	Large
Number of simulations	247	272	473
Success (%)	14.6	82.0	100
Timeliness (weeks)	1.0	1.0	1.0
Se_a_	0.0	30.4	85.7
PPV_a_	0.0	22.2	47.1
NPV_a_	92.9	91.1	98.0
Se_b_	0.0	16.7	14.5
PPV_b_	0.0	16.7	14.7
NPV_b_	100	100	99.8
False alarm rate	0.15	0.16	0.15

Small: < = 100 simulated deaths, Medium: 101–10,000 simulated deaths, Large: > 10,000 simulated deaths.

### Analysis of Sensitivity Linked with Disease Parameters

The median duration of the outbreaks decreased as R_0_ and the daily mortality rate rose because of a faster increase in the proportion of simulated deaths (S1A and S1B Fig). On the other hand, the proportion of infected herds and infected hexagons in simulations were the highest for the scenario with a high R_0_ and a low daily mortality rate (corresponding to the combination of parameters that enhanced the diffusion of the disease in the cattle population) (S1C and S1D Fig).

The sensitivity analysis underlined a significant impact of R_0_ on all performance indicators, but no significant influence of the mortality rate (Table 6, S2 Fig). The global success rate varied from 59 to 87% for simulations with R_0_ values ranging from 3 to 9, slightly increasing as the daily mortality rate increased; for outbreaks simulated with R_0_ of 1, the proportion of success was under 30% (S2A Fig). For most simulations, median timeliness was 1 or 2 weeks; higher delays of detection (equal or above 5 weeks) were predicted for simulation with R_0_ of 1 (whatever the mortality rate) or for R_0_ of 3 combined with a mortality rate of 1% per day (S2B Fig). The detection method was predicted to have null Se_a_ and PPV_a_ for simulations with R_0_ of 1, as a result of low disease diffusion in the population (S1 Fig). On the other hand, the performances of the method improved for higher R_0_, especially when combined to moderate to high mortality rates, with median Se_a_ ranging from 54 to 80% and median PPV_a_ constant around 35–40% (except for R_0_ of 3 combined with a mortality rate of 1% per day) (S2C and S2D Fig). Median NPV_a_ was around 85% for simulations with R_0_ of 1, but ranged between 91 and 98% for higher R_0_ (S2E Fig).

**Table 6:** Sensitivity of model parameters (R_0_ of 1, 3, 5, 7, 9 and daily mortality rate from 1 to 5%) on performance indicators of the cluster detection method.

Indicators	R_0_		Mortality	
	LCC*	p-value^†^	LCC*	p-value^†^
Success	0.72	<0,001	0.29	0.160
Timeliness	-0.73	<0,001	-0.26	0.214
Se_a_	0.81	<0,001	0.28	0.170
PPV_a_	0.67	<0,001	0.06	0.759
NPV_a_	0.84	<0,001	0.21	0.321
Number of simulated deaths at first alert	0.36	0.073	-0.02	0.938
Number of infected herds at first alert	0.01	0.947	-0.78	<0,001
Number of infected hexagons at first alert	0.33	0.105	-0.61	<0,001
Number of simulated deaths per herd at first alert	0.57	0.003	0.72	<0,001

*Linear correlation coefficient (LCC) between input parameter values and median performance indicator.

^†^ Probability of a t-statistic (based on N-2 df) that evaluates LCC = 0

The number of simulated deaths at first alarm was not altered by the characteristics (R_0_ and mortality rate) of the simulated disease (S3A Fig, Table 6). The median number of infected herds and infected hexagons were significantly influenced by the mortality rate, but not by R_0_. More precisely, the median number of infected herds was higher for simulations with a mortality rate of 1%, as was the number of infected hexagons, but was relatively constant for other combinations of R_0_ and mortality parameters (S3B and S3C Fig). The median number of deaths per infected herd increased with R_0_ and mortality rate, with up to 8 dead cattle per infected herd at first alarm for R_0_ of 9 and daily mortality rate of 5% (S3D Fig).

## Discussion

The purpose of our study was to evaluate the performances of an algorithm developed for detecting abnormal increases of deaths in the frame of an automated surveillance system of cattle mortality in France. The evaluation was first conducted by injecting extra deaths caused by a transmissible infectious disease (R_0_ = 5 and daily mortality rate of 3%) comparable to foot-and-mouth disease [26,27]. We then extended the analysis to a large range of alternative epidemiological parameters, characterizing potential diseases that would be targeted by the automated surveillance system, such as bovine tuberculosis (R_0_ ranging from 1 to 5 [34,35]) and infectious bovine rhinotracheitis (R_0_ from 4 to 7 [36,37]).

### Results of the Performances Evaluation

The proportion of successes (i.e. the proportion of trials with at least one true alarm) was 73% among the simulations from the model with a R_0_ of 5 and a daily mortality rate of 3%. As expected, the detection performances varied greatly according to the size of the simulated outbreaks. The proportion of successes was 84.4% when considering only outbreaks causing more than 100 deaths. At the opposite, the algorithm was hardly able to identify outbreaks causing less than 100 deaths. Similarly, the analysis of the sensitivity linked with disease parameters highlighted a low rate of success for outbreaks simulated with a R_0_ of 1 which generated a low number of deaths. The proportion of successes is not very informative by itself: for a disease scenario characterized by a R_0_ of 5 and a daily mortality rate of 3%, half of the simulations caused at least 15,000 extra deaths, which cannot be overlooked since it corresponds to an increase of 5% of the national average weekly death number during 12 weeks. Such an increase in mortality is expected to be detected by the fallen stock companies, as has been seen during the heat wave of 2003 which caused about 8,760 extra deaths [5]. The cumulative proportion of successes is more informative and confirmed that the sensitivity of the system was satisfying: the cumulative proportion of successes was 52% and 72% on week 1 and 4, respectively, when the median number of simulated deaths was only 55 and 112, respectively, on these weeks.

The mean estimate of Se_a_ showed that almost half of the outbreak weeks were flagged with an alarm whereas the value of Se_b_ showed that on average only 13% of the infected hexagons-weeks were included in clusters. These results suggest that once a cluster is identified, the number of infected hexagons will often be greater than the number of hexagons included in the cluster. This low value of Se_b_ could be due to the maximal size of clusters (20% of the population) we set for the scan statistics but this limitation was necessary to make the results of cluster detection useful for policy makers. The low value of Se_b_ should also be linked to the very stringent definition of cases we applied: from one single simulated death, hexagons were considered as infected. Thus a large number of infected hexagons that were not included in a cluster (no detection of the outbreak) may actually contain very few extra deaths. Accordingly, clusters identify hexagon-weeks exhibiting the highest excess mortality; the mean number of simulated deaths per infected hexagons (averaged across simulations) was 76 for outbreak weeks with an alarm versus 13 for outbreak weeks with no alarm. In addition, our algorithm was designed to detect atypical mortality signals in neighboring hexagons only, although the disease was simulated to spread also to distant hexagons through the network of national movements between farms. As a consequence, our algorithm was not designed to detect signals that appeared simultaneously in distant hexagons. Operationally, the system will identify most likely clusters, and follow-up epidemiological investigations of between-herd movements, herd contacts, personnel contacts, etc. will then be needed to trace herds with only few cases and distant outbreaks. A perspective to address this problem could be to design a system where our detection algorithm is not applied at national level, but in each of the regions, which could allow detecting signals appearing in different locations at the same time.

The global timeliness for outbreak detection, that corresponded in our study to the delay between the onset of a signal (increased mortality in a cattle herd) and the generation of an automated alert, was satisfactory: the sensitivity analysis showed that median timeliness varied from 1 to 2 weeks for most scenarios, with a relatively small size of outbreak at first alarm (S3 Fig); yet, higher delays of detection (equal or above 5 weeks) were predicted for slow-spreading diseases (R_0_ = 1). These results suggest that the algorithm could identify an increase in mortality at a stage early enough to allow policy makers to order investigations and organize response, with the limit that other factors affecting the global timeliness of the system (such as the time between the exposure and the first excess deaths, the data collection timeliness, the frequency at which the algorithm is run, etc.) are adequate. Timeline of the death notifications and constraints influencing the notification are fully discussed in Perrin et al. (2012) [6]. In our case the time between the occurrence of a death on a farm and its recording through the system depends on the time elapsed between 1) the death and the discovery of the cadaver by the farmer, which is related to the herd surveillance intensity, and 2) the discovery of the cadaver and its notification to the NCR (which is mandatory within 7 days). An additional delay of one week is required for the control of data quality before analysis. Since 2008, the disposal request, which is required to occur within 48 hours (French Rural Code, Article L226-6) after the discovery of a cattle death, is recorded in the Fallen stock data interchange (FSDI) system within a median time of 2 days [6]. Disposal request data cover the whole French cattle population and are made available more promptly than NCR data. Therefore, they may serve as an appropriate indicator for a timely surveillance system when a sufficiently large historical dataset will be available to model the cattle mortality baseline.

Beside timeliness and sensitivity, specificity is the third main descriptor for outbreak detection performances. Specificity of a detection algorithm can be evaluated on baseline data without injecting any signals [16], with the condition that the baseline did not contain any signals from true outbreaks. This assumption was not applicable to the authentic dataset available for this study, because we did not have information to distinguish real health events from artifacts. We decided to systematically remove outliers from our baseline data and thus could not properly evaluate the specificity of our algorithm. Filtering the baseline also permitted to derive a better evaluation of the real performances regarding sensitivity and timeliness, which could have been affected by the presence of authentic signals in the baseline. Using a smoothed authentic baseline allowed us to test the algorithms in conditions closer to reality than with a fully synthetic baseline. Indeed the smoothed dataset remained close to the initial dataset since the filtering method removed less than 1.3% of the death counts. Theoretically the smoothing did not affect main features of the cattle mortality baseline observed in France, such as important seasonality which varies among regions, long term variations from one year to another, or spatial heterogeneity.

### Design of the Detection Algorithm

We did not directly apply the space-time scan statistics using crude weekly death counts and population estimates, because it would have led to flag clusters of increased mortality rates actually due to the local seasonality of mortality baseline [9]. We addressed this issue, by using adjusted expected counts in line with the approach described in Kulldorff et al. (1997) [31], with the exception that our predictor variable was the time as in Nordin et al. (2005) [38]. Fitting a Poisson model in each hexagon allowed us to adjust expected death counts on local trend and seasonality of mortality rates. We fitted Poisson models in each hexagon as independent units. We did not adjust for the spatial auto-correlation since we were interested in detecting clusters due to such correlation. Considering the number of models fitted, we could not check their validity individually. Yet, Poisson model with a month effect and population as offset has been found to be adequate to predict baseline mortality in French cattle herds from NCR data [9].

### Method Applied for Evaluating the Performances

Using authentic outbreak data for evaluating detection performances is interesting because it demonstrates straightforward the efficiency (or lack of) of a system for a particular event. However it is difficult to consistently or comprehensively identify all outbreaks that occurred during a period of interest [14]. The occurrence and exact timing of true outbreaks in historical data often remain unknown. This was the case for the French 2008–2009 bluetongue epidemics, on which we initially considered to perform our evaluation since we had available a national database with the date and herd number of each official outbreak notification. However we suspected an under-diagnosis of infected herds and animals, particularly at the beginning of the epidemic when farmers and veterinarians were not familiar with the disease, due to its often non-specific clinical signs in cattle [9]. As a consequence, our confidence in location and date of outbreaks was not high enough to consider the official outbreak notifications as gold standard for the occurrence of true outbreaks.

Thus we evaluated the performances of algorithm by simulation, superimposing simulated outbreaks on historical trend data, as it has already been used for syndromic surveillance [38,39]. Outbreaks can be simulated through simple methods (mathematical function such as a step-function or an exponential distribution) or more complex models [40]. To approach realistic conditions and cover a larger range of outbreak types, we did not directly control the outbreaks’ magnitude, duration, spacing, and temporal progression, but simulated the outbreaks through an epidemic model which took into account alternative disease-related factors and the dynamics of the French cattle farm network. As a result, signals were stochastic in space and time, reflecting the variability of real infectious disease dissemination in a cattle farm network. Evaluating performances on numerous and various signals was particularly important since the algorithm evaluated was designed in the frame of a non-specific surveillance system and thus needed to assess the ability of the system to detect a broad range of possible signals.

### Perspectives

The relevance of a mortality-based surveillance system has been questioned in the human health sector as a study conducted on coroner-based mortality surveillance did not lead to satisfying results [16] because of the lack of sensitivity and the limited outbreak detection reliability of the methods studied. In contrast, our findings suggest than an adequate analysis of mortality data could provide satisfactory detection performances to be helpful for surveillance of the cattle population given that an appropriate balance between sensitivity and specificity is found.

Our approach does not allow the identification of a particular herd or group of herds, but flag clusters of 25km-diameter hexagons with abnormal mortality increases. Yet, a second step of analysis completing the presented approach could be to identify herds with the most important excesses of deaths within the hexagons in clusters. We applied our detection algorithm on the whole French territory but the same approach could be applied to restricted areas, such as regions or *departments* (metropolitan France is divided into 22 regions and 96 *departments*). Performing the detection algorithm at an administrative region level may have better performances. Indeed the more precise the alarm (i.e. high sensitivity), the easier and faster the response but also the lower the power of the analysis (i.e. low specificity). Besides, hexagons have no administrative reality which makes the intervention of public forces more difficult to organize practically at national scale when clusters encompass hexagons from several departments or regions. Performing anomaly detection at a smaller administrative scale would be meaningful since each French region and *department* is managed relatively independently by local veterinary services. Should these services be the recipient of alarms, they could more efficiently interpret the signals and carry out the investigations when needed.

Syndromic systems do not target a particular disease but potentially a broad range of health events. Thus the shape, date and magnitude of the signals that such systems should be able to flag are unknown and can range widely. Our analysis predicted relatively good performances of the method of detection for diseases with R_0_ between 3 and 9 and moderate to high mortality rates: a median timeliness of 1 week and a median sensitivity (Se_a_) over 50% (increasing with the number of simulated deaths) for four false alerts per semester. The approach we applied targeted single anomalies (peaks) but other types of anomalies may occur, such as recurrent small (not significant) increases of mortality at the herd level (as in the simulations of the disease scenarios with a R_0_ of 1 which caused only few deaths per herd). Additional temporal methods, such as the CuSUM method (e.g. [41]), which applies the scan statistics on the cumulative sum of deviations of observed from expected counts (residuals), calculated in each hexagon, did not improve detection success (results not shown). Further studies are needed to identify methods able to identify a broader range of signals.

While attaining sufficient timeliness and sensitivity with the evaluated tool seems a reachable purpose, ensuring a sustainable false alarm rate is more difficult. We cannot conclude on the specificity of the system since we do not know which proportion of the atypical observations we removed from the initial dataset were artifacts and how many corresponded to real health events. Most outliers removed corresponded to the occurrence of a high number of deaths in hexagons where there were historically no or very few deaths (e.g. 140 deaths occurring in the hexagon 840 on week 2009-10-01 when 45 were expected). Many of them were probably related to a real event, but not necessarily of epidemiological interest, as for example the culling of an entire herd, an animal transporter crash or a fire on a farm. In the frame of an operational system, if the user does not want to yield alarms for such events, then it could be necessary to treat this kind of death notifications differently, for example by changing the notification system. It would also be possible to adjust the mortality rate predictions on other factors that may not be of interest for the users. For example using bio climatic factors in the predictions model would allow no detection of mortality increases due to an unusual heat wave or cold snap [42].

## Conclusion

Our study suggests that the proposed approach combining temporal regression and cluster detection applied to the data from the national cattle register could help identifying automatically unusual excess deaths occurring in the French cattle population. Results of the simulation study are encouraging, but the real performances of such a system for detecting outbreaks still need to be challenged. We suggest testing the efficiency of the approach by prospectively evaluating an operational pilot system whose performances would be routinely investigated and recorded (number of real outbreaks detected, false alarms, and outbreaks missed or detected late). Such an approach would moreover answer the question whether (and in which cases) abnormal mortality increases can be timelier signals than the traditional notification of unusual clinical situations from field professionals, farmers or veterinarians.

The proposed automated surveillance system is not meant to replace the tight network of veterinarians directly in contact with the field, which constitutes the core of every animal health surveillance system. It should rather be used as an adjuvant in conjunction with other surveillance activities. Accordingly, we believe that our detection tool could globally increase the probability of detecting unusual health events and facilitate the rapid implementation of adapted control measures by animal health services.

## Supporting Information

S1 Fig Characteristics of outbreaks simulated with reproduction ratios R_0_ ranging from 1 to 9 and daily mortality rate from 0.01 to 0.05. Planes represent the first quartile (dark grey), median (medium grey) and third quartile (light grey) for each descriptor of the simulated outbreaks. Note that the order of the values on the axes for R_0_ and the daily mortality rate varies among graphs.
(TIFF)

S2 Fig Analysis of the sensitivity of performance indicators to the two main parameters of the SIR model, R_0_ and the daily mortality rate. Planes represent the first quartile (dark grey), median (medium grey) and third quartile (light grey). Note that the order of the values on the axes for R_0_ and the daily mortality rate varies among graphs.
(TIFF)

S3 Fig Characteristics of outbreaks simulated with reproduction ratios R_0_ ranging from 1 to 9 and daily mortality rate from 0.01 to 0.05 at first alarm. Planes represent the first quartile (dark grey), median (medium grey) and third quartile (light grey). Note that the order of the values on the axes for R_0_ and the daily mortality rate varies among graphs.
(TIFF)

S1 File Details of the SIR model (simplified from Rautureau et al., 2012). (DOCX)
