# Supplementary figures and images for: Simulation-Based Evaluation of the Performances of an Algorithm for Detecting Abnormal Disease-Related Features in Cattle Mortality Records
Source: PLoS One. 2015 Nov 4;10(11):e0141273. doi: 10.1371/journal.pone.0141273 (PMC4633029; doi:10.1371/journal.pone.0141273)

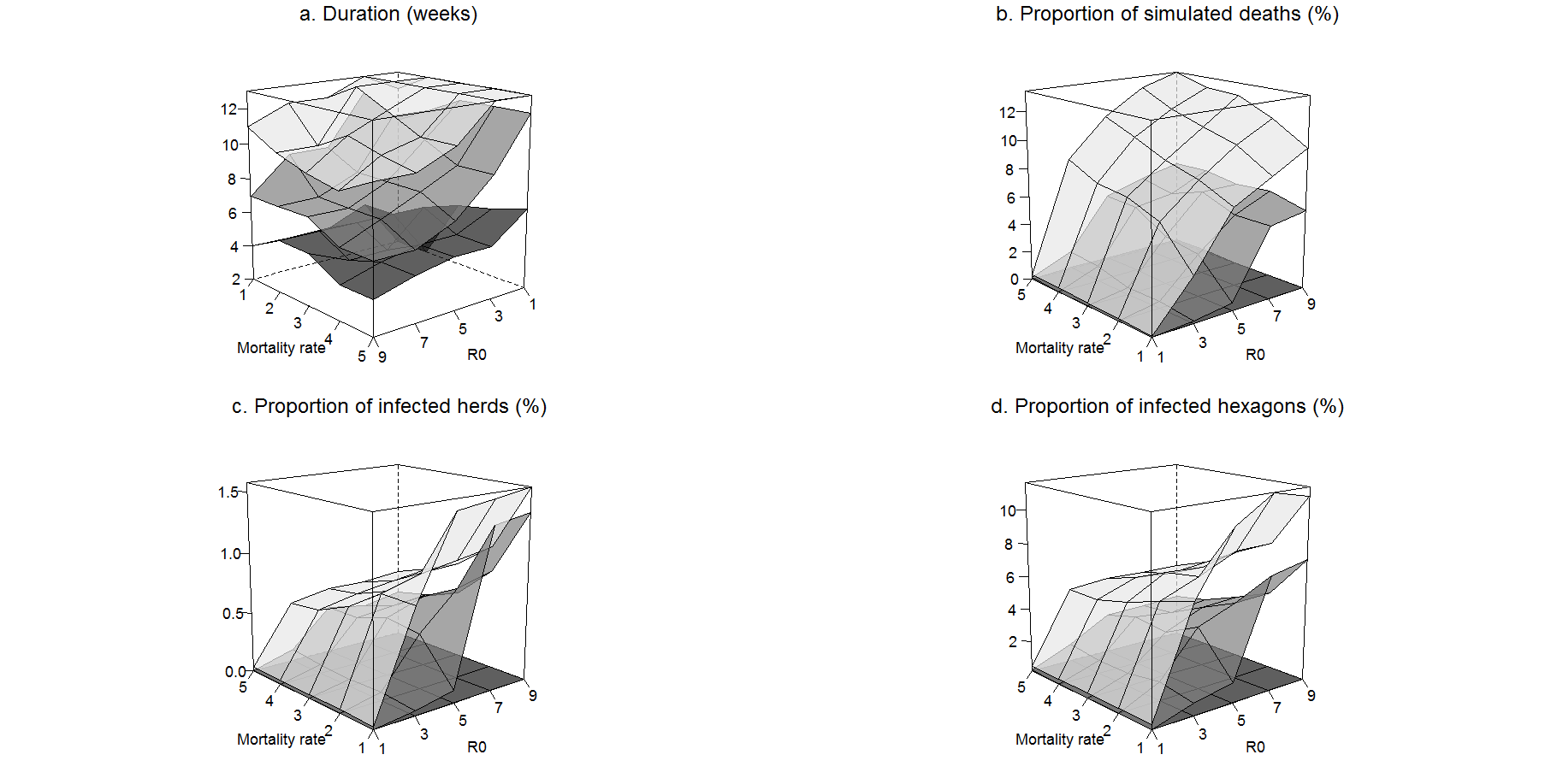

Supplement: S1 Fig — Planes represent the first quartile (dark grey), median (medium grey) and third quartile (light grey) for each descriptor of the simulated outbreaks. Note that the order of the values on the axes for R0 and the daily mortality rate varies among graphs. (TIFF) [file pone.0141273.s001.tiff]

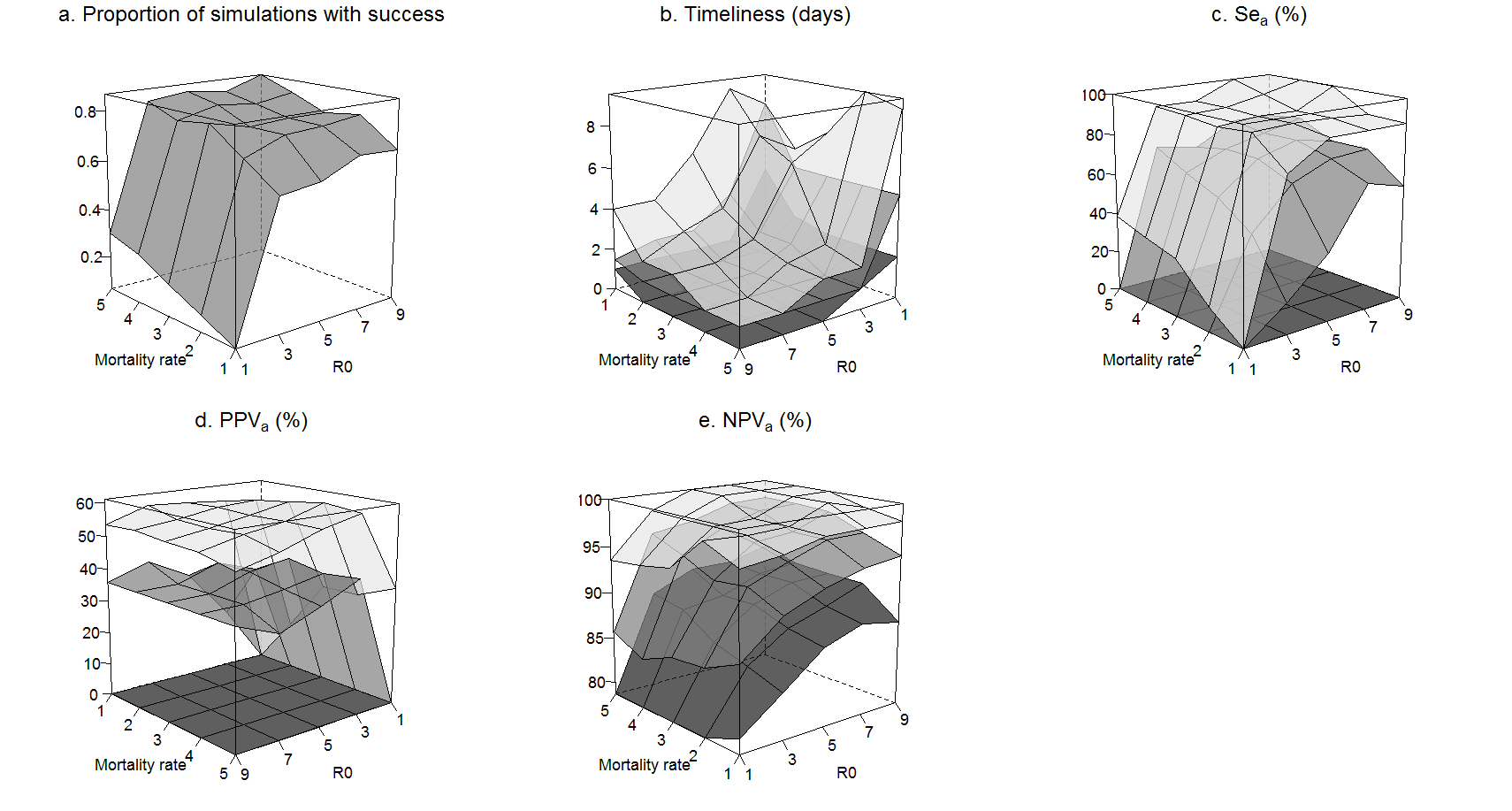

Supplement: S2 Fig — Planes represent the first quartile (dark grey), median (medium grey) and third quartile (light grey). Note that the order of the values on the axes for R0 and the daily mortality rate varies among graphs. (TIFF) [file pone.0141273.s002.tiff]

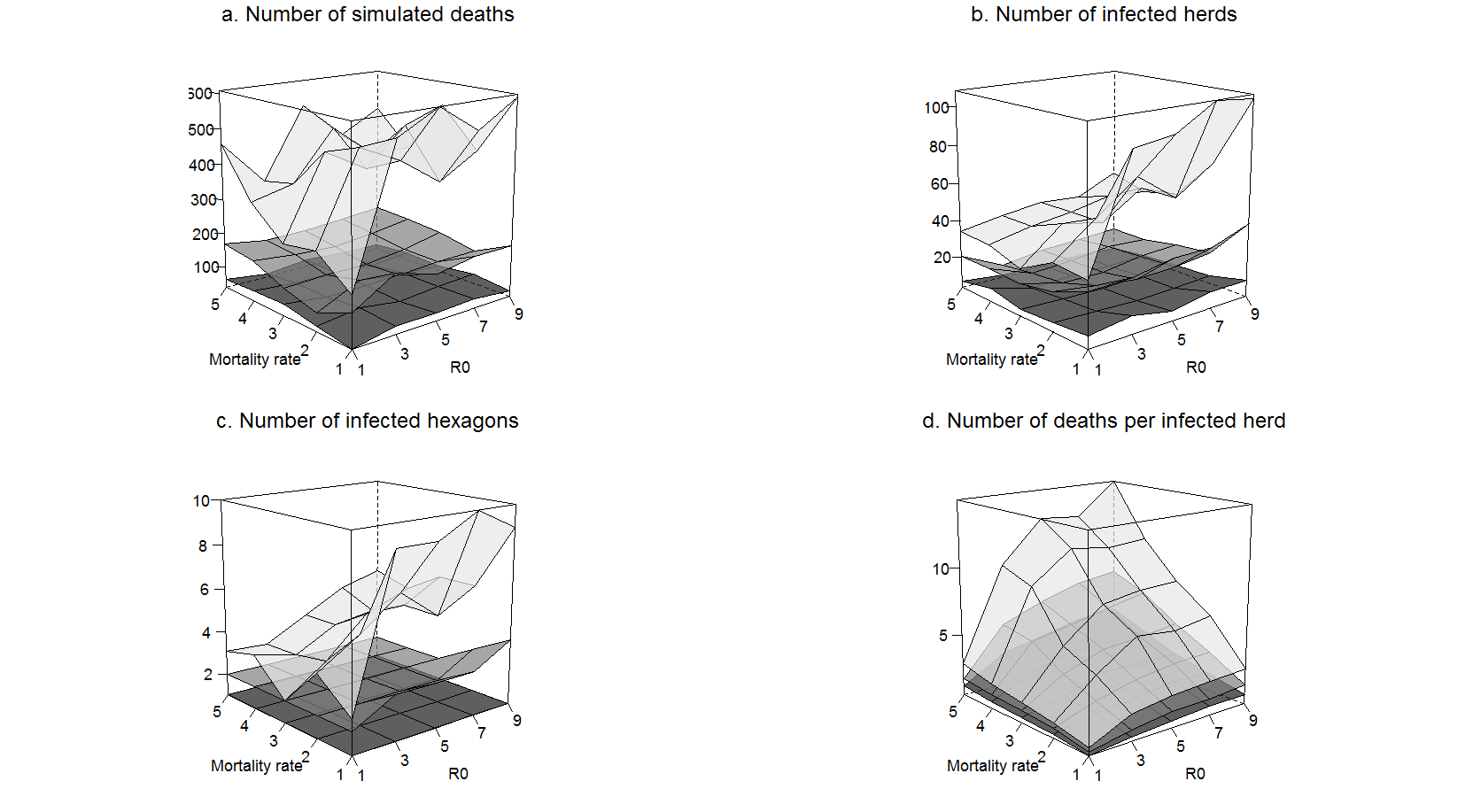

Supplement: S3 Fig — Planes represent the first quartile (dark grey), median (medium grey) and third quartile (light grey). Note that the order of the values on the axes for R0 and the daily mortality rate varies among graphs. (TIFF) [file pone.0141273.s003.tiff]
