# Supplementary material for: Simulation-Based Evaluation of the Performances of an Algorithm for Detecting Abnormal Disease-Related Features in Cattle Mortality Records
Source: PLoS One. 2015 Nov 4;10(11):e0141273. doi: 10.1371/journal.pone.0141273 (PMC4633029; doi:10.1371/journal.pone.0141273)
Supplement: S1 File — (DOCX) [file pone.0141273.s004.docx]

S1 File.

A stochastic state transition model was used with a daily time step. The basic unit was the herd. Three individual exclusive health states were considered: exposed and susceptible (denoted *S*), infectious (*I*), and recovered (*R)*. At a given time step, the sizes of the corresponding sub-populations in herd *i* were denoted as: *S_i_*, *I_i_*, *R_i._* Similarly, the number of animals in herd *i* was denoted *N_i_*.

Disease transmission was modeled to occur within herds and between herds through both live animal movements and direct transmission by contacts between herds. The probability for an animal in the state *S* on the herd *i* of getting infected by a direct contact from an animal in herd *i* or in herd *j* was:

$$p_{inf}=1-e^{-\left[\beta\frac{I_{i}}{N_{i}}+\left(1-\phi\right)\sum_{j\in V_{i}} \beta\frac{I_{j}}{N_{j}}\right]}$$

where β is the within-herd transmission parameter (0.86), φ is the biosecurity parameter influencing the between-herd transmission (0.75) and *V_i_* is the neighbors of the herd i in the network of direct contact, defined by the function:

$$I_{j\in V_{i}}\sim Bern\left(e^{-Kd\left(i,j\right)}\right)$$

where *Bern*(*p*) is the Bernouilli distribution of parameters *p*, *d*(*i*, *j*) is the Euclidian distance between herds *i* and *j*, and *K* is the parameter for the transmission kernel (*K*=0.002). The transmission from herd *j* to *i* was thus modeled by the fraction of the force of infection in herd *j*, with a proportionality factor based on the biosecurity parameter in herd *i*.

Between-herd transmission by commercial transfers of animals was simulated mechanically at each time point by random sampling of the state of the animals that are moved from a herd to another.

The probability of dying for an animal in the state *I* in the herd *i*:

$$p_{mort}=\left[1-\left(1-\alpha\right)\left(1-\mu\right)\right]\frac{\mu}{\mu+\alpha}$$

where α the daily recovery rate of infected animals (0.14) and μ the daily mortality rate of infected animals (0.03).

The probability of getting immunized for an animal in the state *I* in the herd *i* was:

$$p_{immun}=\left[1-\left(1-\alpha\right)\left(1-\mu\right)\right]\frac{\alpha}{\mu+\alpha}$$

For each day, *n_i,S-I_*, *n_i,I-M_*, *n_i,I-R_* were the numbers of animals in the state I that get infected, die, or get immunized:

$$n_{i,S-I}\sim Bin\left(S_{i},p_{inf}\right)$$

$$n_{i,I-M}\sim Bin\left(I_{i},p_{mort}\right)$$

$$n_{i,I-R}\sim Bin\left(I_{i},p_{immun}\right)$$

where *Bin*(*n*, *p*) is a binomial law of parameters *n* and *p*.

Parameter values were derived from Rautureau et al. (2012).

Reference

Rautureau, S., B. Dufour, and B. Durand. 2012. Structuring the passive surveillance network improves epizootic detection and control efficacy: a simulation study on foot-and-mouth disease in France. Transbound Emerg Dis 59:311-322.
